# Cardiogenic shock following cardiac tamponade and Takotsubo in COVID-19

**DOI:** 10.2217/fca-2020-0115

**Published:** 2020-10-20

**Authors:** Asad J Torabi, Josue Villegas-Galaviz, Maya Guglin, Kyle Frick, Roopa Rao

**Affiliations:** ^1^Division of Cardiology, Krannert Institute of Cardiology at Indiana University School of Medicine, 1800 N Capital Avenue, Indianapolis, IN 46202, USA; ^2^Department of Internal Medicine, Indiana University School of Medicine, 1120 W Michigan St, Indianapolis, IN 46202, USA

**Keywords:** cardiac tamponade, cardiogenic shock, COVID-19, heart failure, Takotsubo

## Abstract

**Introduction:** Takotsubo is often described as stress-induced cardiomyopathy and is a known cause of heart failure. **Objective:** Review the clinical course of a young coronavirus disease 2019 (COVID-19) patient who developed Takotsubo following cardiac tamponade. **Case presentation:** A 42-year-old woman presented to the emergency department with fever, altered mental status and hypoxia. She was ultimately found to be in cardiac tamponade and within 2 hours of a pericardiocentesis she developed Takotsubo and was in cardiogenic shock. Her family decided to place her on comfort measures and she died the same day. **Discussion:** This case illustrates the increasing number of cardiovascular complications being reported in COVID-19 and highlights the importance of clinicians to be aware of these challenges. **Conclusion:** Here, we report a distinct presentation of cardiogenic shock in a young COVID-19 patient. The rapid onset of her suspected Takotsubo and the severity of her disease were striking features in this case.

Takotsubo cardiomyopathy is a reversible cardiomyopathy with a dramatic presentation, mimicking acute myocardial infarction [1]. The echocardiogram of typical Takotsubo is characteristically described as a left ventricle with hyperkinetic basal segments and hypokinetic/dyskinetic/akinetic mid and apical segments [2]. This process was first described in Japan in 1990 and it was not until 2006 when Takotsubo was formally identified as an acquired form of cardiomyopathy [3]. Clinical presentation of Takotsubo can have overlapping features with myocarditis. The role of imaging has helped in this regard. Apart from characteristic wall motion abnormalities on echocardiography [4], mitral regurgitation (systolic anterior motion or tenting), dynamic left ventricular (LV) outflow track obstruction from hyperdynamic basal segments or ventricular thrombus are associated findings. Cardiac MRI is both complementary and confirmatory in helping to characterize Takotsubo [5]. In Takotsubo, there tends to be edema but no hyperenhancement with gadolinium. Acute myocarditis often reveals a patchy hyperenhancement on MRI.

As the COVID-19 pandemic has progressed, there has been a higher incidence of Takotsubo when compared with controls from 2018 to 2019. In a retrospective analysis, the length of stay in the hospital has been longer for these patients, but no difference in mortality or hospitalization [6]. Here, we report the case of a 42-year-old woman with COVID-19 who presented with cardiac tamponade and developed cardiogenic shock from suspected Takotsubo cardiomyopathy after pericardiocentesis.

## Case presentation

A 42-year-old female presented from a nursing home with 1 week of worsening mental status and fever. Past medical history was notable for having Crohn’s disease on vedolizumab and Guillain–Barré syndrome (GBS) 6 months prior to presentation. She had no known cardiovascular risk factors. Physical exam on arrival to the emergency department was pertinent for a blood pressure of 93/62 mmHg, heart rate of 139 beats/min, temperature of 38.2°C and an oxygen saturation of 89% on 15 l/min. She had diffuse crackles on lung exam and no cardiac murmurs.

**Figure 1.**
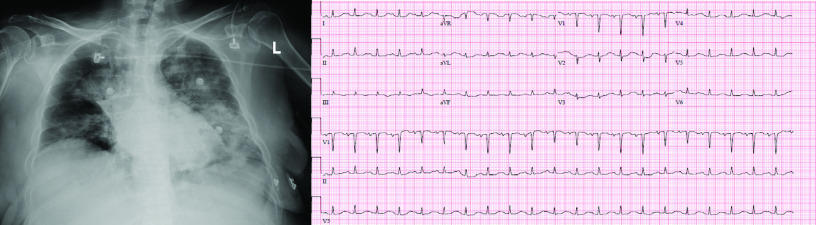
Chest x-ray and ECG. Chest x-ray (left) with pulmonary infiltrates seen in COVID-19. ECG (right) with low voltage in limb leads.

Chest x-ray demonstrated a normal cardiac silhouette with patchy consolidative opacities in the lung fields and electrocardiography revealed low voltage in the limb leads (Figure 1). Laboratory testing showed a cardiac troponin-I of 0.29 ng/ml, B-type natriuretic peptide of 612 pg/ml and white blood cell count of 9.2 k/mm^3^. Initial transthoracic echocardiography revealed a hyperdynamic left ventricle and a hemodynamically significant moderate-sized pericardial effusion with right atrial systolic collapse (Supplementary Video 1). There was also mild variability between the mitral valve and tricuspid valve inflow velocities. Despite aggressive fluid resuscitation, the patient was persistently hypotensive. She emergently underwent pericardiocentesis with only 65 ml of serous fluid removed. A limited transthoracic echocardiography was repeated within 2 h for persistent hypotension on multiple pressors (levophed, vasopressin and phenylephrine). There was no significant residual pericardial effusion, however, her LV ejection fraction had decreased significantly to 20%. The LV apex was dilated with systolic hypokinesis and basal segments had preserved contraction supporting the diagnosis of Takotsubo cardiomyopathy. (Supplementary Video 2). Pericardial fluid was sent for bacterial culture, acid fast bacilli smear and fungal culture which all resulted negative. Serum inflammatory makers were elevated: C-reactive protein (CRP) 14.7 mg/dl, ferritin 310.1 ng/ml, D-dimer was 226 ng/ml and IL-6 was 2362 pg/ml. Peak troponin was mildly abnormal at 0.86 ng/ml.

The etiology and differential diagnosis of shock in a patient with altered mental status, hypoxia and fever is wide. In an immunocompromised patient, from a nursing home COVID-19 infection was high on the differential especially in the setting of pandemic, shock could be distributive due to sepsis and or cardiogenic. Differential diagnoses for cardiogenic shock included, fulminant viral myocarditis, cardiac tamponade, pulmonary embolism and acute congestive heart failure.

The patient was started on broad spectrum antibiotics for acute respiratory distress syndrome from fulminant pneumonia. Patient underwent left heart catheterization for severely reduced ejection fraction, which showed normal coronaries and mildly elevated left ventricular end diastolic pressure. An intraaortic balloon pump was placed as a bridge for maximal support. For persistent hypotension vasopressors were cautiously used including norepinephrine, vasopressin and phenylephrine. She continued to have refractory hypoxia with pulse oxymetry in 70s to 80s despite ventilator optimization (positive expiratory pressure of 12 cm H_2_O, FiO2 100%). At this point she was transferred to a tertiary care center for consideration of advanced circulatory support. She was determined not a candidate for veno-arterial extracorporeal membrane oxygenation support because of her overall poor functional status prior to presentation and prognosis following her GBS. She was closely monitored in the intensive care unit for the next 48 h. During that time her COVID-19 PCR came back positive. Her clinical condition continued to deteriorate with maximum ventilator support and developed multiorgan failure with anuric renal failure and bowel ischemia. Due to overall poor prognosis, she was placed on comfort measures after discussion with the family and she passed away the same day. Consent was obtained for the procedures (cardiac catheterization, intraaortic balloon pump and pericardiocentesis).

## Discussion

The Mayo Clinic diagnostic criteria for Takotsubo is widely used to aid in the diagnosis of Takotsubo [1]. Features in favor of Takotsubo in our case includes, characteristic appearance of the echocardiogram showing hyokinesis of the LV mid and apical segments, absence of the obstructive coronary artery disease, and a modest rise in troponin which peaked within 24-h [7]. We were not able to repeat another echocardiogram to demonstrate whether this hypokinesis was transient as the patient died within 48 h and also the need to limit the amount of personnel and testing to decrease the exposure of healthcare workers. Electrocardiographic findings are variable in Takotsubo including a normal ECG. However, the Mayo diagnostic criteria list ST-segment elevation or T-wave inversions as points in favor of Takotsubo. Cardiac magnetic resonance would have been helpful in distinguishing myocarditis from Takotsubo; however, this was not performed because our patient was quite unstable and also the echocardiogram was characteristic of Takotsubo. We also did not screen for pheochromocytoma.

Cardiovascular complications from COVID-19 are being increasingly recognized [8], along with concern for lasting cardiac consequences [9]. We believe our patient’s pericardial effusion developed as a sequela of her COVID-19 infection. This is a plausible speculation as other viral infections can lead to pericarditis complicated by pericardial effusions [10]. This mechanism in COVID-19 leading to pericardial efffusion is yet to be defined but is a likely consequence of an inflammatory cascade [11]. We suspect the pericardial effusion in our patient may have accumulated rapidly given her relative hemodynamic instability with moderately sized pericardial effusion, requiring pericardiocentesis.

As more time has progressed during the COVID-19 pandemic, there have been more emerging cases of Takotsubo and COVID-19 [6,12–14]. Our case was distinctive because of the severity of disease and impressive rapid progression to suspected Takosubo following pericardiocentesis in less than 2 h. In a small five patient series of cases with COVID-19 and Takotsubo [14], all patients were male, two resulted in death and only one was associated with pericardial effusion. The troponin-I peak was 11.4 ng/ml, CRP 207 mg/dl, IL-6 56 mg/dl and ferritin peak 1946 ng/ml, and these markers were significantly higher than the cases with no cardiac injury. All the five cases had features suggestive of Takotsubo by echocardiography and none of these patients were evaluated with cardiac catherization. Generally, the prognosis of Takotsubo tends to be favorable [15]; however, our patient ultimately went into multi-organ failure and mixed shock.

The pathophysiology of Takotsubo remains uncertain. Proposed mechanisms include the adrenergic stress hypothesis, epicardial coronary dysfunction, coronary microvascular dysfunction, brain–heart interactions, oxidative stress and estrogen depletion. Stress is thought to lead to catecholeamine release and myocardial injury. Multitudes of triggering event and female preponderance are commonly noted [16]. A few proposed mechanisms of Takotsubo in COVID-19 are cytokine-mediated injury, viral myocardial infiltration and microvascular thrombi [17]. A few counfounding factors in our case make it difficult to know if COVID-19 was the single underlying cause that may have led to Takotsubo. There were other potential sources of stress from procedures, such as intubation or pericardiocentesis, which may have occurred. High dose of levophed as an etiology was also in the differential [18]. Higher levels of inflammatory mediators from autoimmune responses, such as from her Crohn disease or GBS, if relapsed, may also have played a role. There are laboratory markers available to measure the extent of injury from COVID-19, such as CK-MB, CRP, IL-6 and ferritin. In myocarditis-like syndromes with or without hemodynamic instability, troponin elevation in the absence of coronary artery disease has been reported as a relatively common manifestation of severe COVID-19 infection [19]. Troponin elevation was reported in 20–30% cases of severely ill patients in China [20,21].

There is still not enough data to suggest an evidence-based approach in treatment of Takotsubo [22]. Congestive heart failure therapy with diuretics, ACE inhibitors and beta blockers are commonly used. Patients with cardiogenic shock are often managed with advanced circulatory support. Inotropic agents are typically avoided or used with caution for fear of causing a left ventricular outflow track obstruction, a dreaded consequence of Takotsubo. Anticoagulation is typically reserved for LV thrombus, which is an infrequent finding.

## Conclusion

Here, we describe a distinct presentation of shock in a young woman with severe COVID-19 infection, which was complicated by a constellation of problems. This case report demonstrates that Takotsubo should be considered in the differential in patients with COVID-19 who present with myocardial damage. The exact pathophysiology of myocardial injury in these patients is not well understood. Inflammatory markers may be helpful in that these tests will likely be significantly more elevated in cases with myocardial injury. While a more extensive work up may be helpful in distinguishing Takotusubo from myocarditis, this may not always be possible due to the severity of the patient’s illness or the need to conserve personal protective equipment.

**Summary points**

- An increasing number of cardiovascular complications have been reported in COVID-19 patients, including cardiac tamponade and Takotsubo cardiomyopathy.
- Cardiac biomarkers have reportedly been elevated in almost a third of severely ill patients in China.
- The pathophysiology of Takotsubo cardiomyopathy remains ambiguous and the diagnosis can be difficult to distinguish clinically from viral myocarditis.
- Common ECG findings seen in Takotsubo cardiomyopathy and viral myocarditis may not always be seen.
